# Bioactive Compounds from Banana Leaf Extracts: Influence of Extraction Methodologies and Their Integration into Knitted Hemp Fabrics

**DOI:** 10.3390/ma17235884

**Published:** 2024-11-30

**Authors:** Joana Mota Gomes, João Mariz, Catarina Rodrigues, Ana Luísa Alves, Joana Moreira, Bárbara Vieira, Rosa Maria Silva, Andrea Zille, Carla Joana Silva

**Affiliations:** 1 CITEVE—Technological Centre for Textile and Clothing of Portugal, 4760-034 Vila Nova de Famalicão, Portugal; jmgomes@citeve.pt (J.M.G.); jmariz@citeve.pt (J.M.); cfrodrigues@citeve.pt (C.R.); rmaria@citeve.pt (R.M.S.); 2 CeNTI—Centre for Nanotechnology and Advanced Materials, Rua Fernando Mesquita 2785, 4760-034 Vila Nova de Famalicão, Portugal; lalves@centi.pt (A.L.A.); jmoreira@centi.pt (J.M.); 3 Centre for Textile Science and Technology (2C2T), University of Minho Campus de Azurém, 4800-058 Guimarães, Portugal; barbaravieira98.bv@gmail.com (B.V.); azille@det.uminho.pt (A.Z.)

**Keywords:** bioactive textiles, banana leaves, hemp

## Abstract

This study explores the bioactive potential of banana leaf extracts and their innovative integration into knitted hemp fabrics. To obtain the extracts, distinct extraction methodologies were employed, namely conventional extraction, ultrasound-assisted extraction, and pressurized-liquid extraction. Aqueous and hydroethanolic solvents, namely 20% (*v*/*v*) and 50% (*v*/*v*), were employed during the extraction process. Furthermore, the cationization and functionalization of knitted hemp fabrics with the banana leaf extracts was achieved through padding. The extracts’ phenolic content and antioxidant activity were evaluated using the Folin–Ciocalteu (FC) and 2,2-diphenyl-1-picrylhydrazyl (DPPH) assays, respectively. The results indicated that both ultrasound-assisted extraction and pressurized-assisted extraction substantially enhanced the yield of phenolic compounds in comparison to conventional extraction, while employing 50% EtOH as a solvent also improved extraction yields for all extraction methodologies. The functionalized knits were further characterized concerning their antioxidant activity by DPPH, assessing their antimicrobial properties through ATCC TM-100 standard against three microorganisms (*Staphylococcus aureus*, *Candida Krusei*, and *Candida albicans*), and UV protection according to the standard AS/NZS 4399:2017. Antioxidant activity was highest in knits functionalized with extracts obtained via ultrasound-assisted extraction, while antimicrobial properties were most pronounced in knits treated with hydroalcoholic extracts, particularly those derived from assisted methods. The UV protection was enhanced in extracts with higher ethanol concentrations obtained through ultrasound-assisted extraction, with these knits exhibiting the highest Ultraviolet Protection Factor (UPF). This research not only highlights the efficacy of the alternative extraction technologies but also offers valuable insights for the development of innovative, biocompatible materials with enhanced bioactive properties for diverse applications in the textile and healthcare sectors, paving the way for sustainable applications.

## 1. Introduction

The large-scale cultivation and production of fruits result in the generation of significant quantities of horticultural residues, such as leaves, peels, seeds, and pulp. Bananas are among the most consumed fruits worldwide, totaling about 119 million tonnes in terms of global production [1,2]. Banana plants were primarily grown for their fruit, and their subsequent application for wine production and as a source of natural fibres [3] has revealed a wealth of untapped potential beyond these conventional uses. In particular, banana leaves have garnered attention across various fields due to their versatility, practical applications, and notable bioactivity. This residue, which is abundantly available and disposed of without proper usage, has been widely studied for the development of packaging solutions [4,5]. The packaging industry has been focusing on the development of biodegradable packaging materials to replace the currently used ones, including polyester, polycarbonate, and polyvinylchloride [4]. Banana leaves and derivatives, obtained through the incorporation of different functional groups (e.g., phosphate, sulphate, and phosphosulfonate), have also been explored for the development of strategies aiming at the removal of ions from aqueous solutions, including Zn (II), Pb (II), and Fe (III), as well as the removal of gaseous pollutants, with efficiencies of 76.2% [4,5,6]. Moreover, banana leaves and banana leaf extracts have attracted considerable attention in the cosmetic and healthcare industries, mostly owing to their rich bioactive compounds. Banana leaves are used for the treatment of skin injuries, including wounds, eczema, irritation, rashes, and sunburn, owing to their cooling effect [3]. The potential of banana leaf extracts obtained through alkali and acetone extractions as a source of pigments has been investigated for dyeing cotton substrates, aiming to enhance the sustainable and eco-friendly nature of textile colouring processes [7]. Banana leaves’ aqueous and hydroethanolic extracts have reported antioxidant and antimicrobial properties [1,3,8,9].

The present work aims to explore the potential of banana leaves as a sustainable resource in enhancing the functionality of knitted hemp fabrics. Hemp fibres are natural, versatile, and sustainable, and have emerged as a promising contender in textiles. Renowned for their robustness and durability, hemp fibres are derived from the stalk of the *Cannabis sativa* plant, which requires minimal water and pesticides to cultivate. This inherent resilience makes hemp a highly eco-friendly alternative to conventional textile materials like cotton [10]. Additionally, hemp possesses exceptional breathability and moisture-wicking properties, ensuring comfort and wearability in various climates. Its natural resistance to mould, UV rays, and abrasion further encourages its suitability for a wide range of textile applications including knitted underwear, t-shirts, and even technical textiles, including those tailored for military use [11,12]. Moreover, as a renewable resource, hemp aligns seamlessly with the global movement towards more environmentally conscious and sustainable practices. Its incorporation into textiles not only reduces the industry’s ecological footprint but also offers consumers a more planet-friendly choice without compromising on quality.

Herein, banana leaf extracts will be obtained through aqueous and hydroethanolic extraction processes at 50 °C, employing conventional, ultrasound-assisted, and pressurized methods. The extracts will be evaluated for their total phenolic content and antioxidant activity and integrated into the knitted fabrics. We anticipated that the proposed approach would confer distinctive attributes to the knitted fabrics, including antioxidant properties, increased UV protection, and antimicrobial activity. At the same time, an evaluation will be conducted to determine the dyeing potential of the extracts on the knitted hemp fabrics. This study aims to pave the way for innovative and eco-conscious applications in textiles by harnessing the inherent properties of both banana leaves and hemp-based fabrics.

## 2. Materials and Methods

### 2.1. Chemicals and Reagents

All chemicals and reagents used were of analytical grade, including 2,2-diphenyl-1-picrylhydrazyl (DPPH) free radicals and ethanol (EtOH), and were purchased from Sigma Chemical Co. (St. Louis, MO, USA). Folin–Ciocalteu reagent was bought from Panreac Applichem (Darmstadt, Germany) and Sodium Carbonate anhydrous from Acros Organics (Geel, Belgium).

### 2.2. Plant Material Preparation

**Figure 1:**
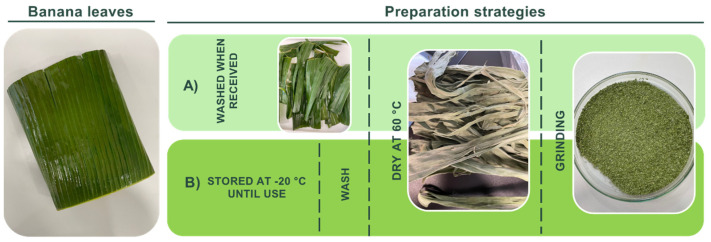
A schematic representation of the strategies used for the preparation of the plant material: (**A**) the samples were received, washed, and dried immediately upon arrival, while (**B**) the leaves were received and stored at −20 °C; when needed, they were defrosted, washed, and dried.

The banana leaves (*Musa sapientum*) used in this study were obtained from *Frutlove*, originally from Thailand. After receiving them, they were either cleaned with cold running tap water to minimize nutrition loss and then dried at 60 °C (strategy A) or frozen at −20 °C upon delivery (strategy B). Figure 1 summarizes the different approaches. After drying, the granulometry of the banana leaves was adjusted to particles ≥ 1 mm, using the SM 300 RETSCH (Hann, Germany) cutting mill.

### 2.3. Moisture Content Determination

The drying of the samples was carried out at 60 °C for approximately 48 h, or until dry-weight stabilization. Based on prior research, it was determined that a temperature of 60 °C would be appropriate, as this study revealed that approximately 90% of the antioxidant activity diminished when the drying temperature was 75 °C [13]. The samples were weighed before drying (*W_i_*) and after drying (*W_f_*), and the dry weight was determined using Equation (1):

(1) $$Dry\;weight\;(\%)=100-\left(\frac{w_{i}-w_{f}}{w_{i}}\right)*100$$

### 2.4. Extract Preparation

For the preparation of the extracts, different strategies were carried out, including the use of different solvents (water and different hydroethanolic solutions) and different extraction methodologies, such as conventional, ultrasound-assisted, and pressurized-assisted extractions. For each extraction, 3% (*w*/*v*) of banana leaf powder was mixed with the respective extraction solvent (water, 20% (*v*/*v*) EtOH or 50% (*v*/*v*) EtOH).

#### 2.4.1. Conventional Extraction

Conventional extractions were performed using the Mathis equipment (MathisLOBOMAT, Oberhasli, Swiss). The assays were performed for 30 min, at 50 °C, with a heat rate of 2 °C/min at a continuous rotation of 25 rpm. After extraction, the mixtures were vacuum-filtered and then centrifuged at 10,000× *g* for 10 min (Centurion Scientific, C4000, Worthing, West Sussex, UK). The supernatant was collected, vacuum-filtered, and stored at 4 °C for further analysis.

#### 2.4.2. Ultrasound-Assisted Extraction

Ultrasound-assisted extraction was carried out using an ultrasonic device operating at low frequencies (20 kHz) with a 1000 W ultrasonic processor UIP1000hdT (Hielscher Ultrasonics, GmbH, Teltow, Germany). The transducer was customized with a BS4d22 standard titanium sonotrode (frontal area: 3.8 cm^2^). The sonication probe, acting as a wave amplifier, was plunged into a beaker containing dried banana leaves (obtained from strategy (B)) previously mixed with proper extraction solvent. The acoustic energy delivered was based on previous works and settled as 6.6 kJ/L with an acoustic amplitude of 35 µm. During ultrasound exposure, the mixture’s temperature was monitored and limited to 50 °C. The extraction was performed under continuous stirring. After the ultrasound treatment, the mixture was incubated at 50 °C for 30 min under continuous stirring and protected from the light.

Finally, the obtained extracts were centrifuged at 12,500× *g* for 15 min (Thermo Scientific™ centrifuge model Sorvall™ LYNX 6000, Waltham, MA, USA) and the resulting supernatants were filtered using 5–13 µm qualitative filter paper and stored at 4 °C for further analysis.

#### 2.4.3. Pressurized-Liquid Extraction

Pressurized-liquid extraction was performed using a 1500 mL Parr batch reactor model 5100 (Parr Instrument Company, Moline, IL, USA).

The reactor was equipped with a stainless-steel jacketed vessel, pressure gauge, mechanical stirrer, adjustable speed impeller, and a thermocouple, and connected to controllers. The extraction vessel was filled with 500 mL of dried banana leaf (obtained from strategy (B)) suspension prepared in the respective solvent extraction. The extraction vessel was then heated at 50 °C and purged with N_2_ to pressurize the vessel to 5 bar. A mixing speed of 300 rpm and a total extraction time of 30 min were used for all extractions. The resulting extracts were centrifuged at 12,500× *g* for 15 min and the supernatants were filtered using 5–13 µm qualitative filter paper and stored at 4 °C for further analysis.

### 2.5. Knitted Fabric Preparation

#### 2.5.1. Knitting

**Figure 2:**
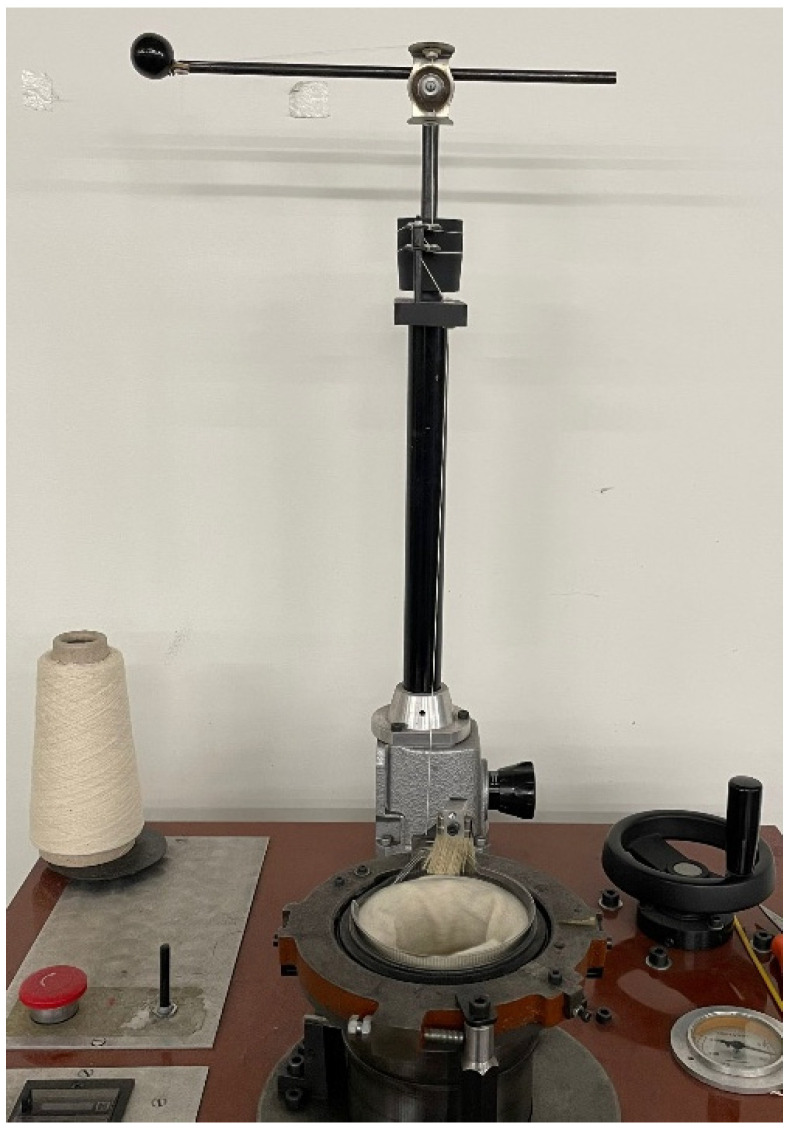
Laboratory-scale Tricolab.

The textile samples were knitted using hemp yarns in a circular laboratorial scale equipment, named Tricolab (Figure 2), with a loop length of 4.0.

#### 2.5.2. The Pretreatment and Functionalization of the Produced Knitted Fabrics: Padding Method

Prior to impregnation with the extracts from banana leaves, the knitted hemp fabrics were subjected to a cationization pre-treatment. Firstly, a solution of 0.5% (*w*/*v*) chitosan in a 0.6% (*v*/*v*) solution of acetic acid was prepared. After, the knitted fabrics were soaked in the 0.5% (*w*/*v*) chitosan solution, passed through a foulard, and squeezed by a pair of rollers with 1 bar of pressure and 80% pick-up. The fabrics were left to dry at 50 °C for 30 min, and then cured, at 150 °C, for 5 min. Following the pre-treatment, the samples were functionalized with banana leaf extracts using the same procedure. Here, the drying was performed at 40 °C, for 20 min, to prevent the bioactive compounds’ deterioration. It is important to note that we did not prepare the knits by boiling or performing half-bleaching, as we used bleached hemp yarn.

### 2.6. The Characterization of the Extracts and Knitted Fabrics 

#### 2.6.1. Total Phenolic Content Determination (Folin–Ciocalteu Method)

The total phenolic content (TPC) of the banana powder residue and the extractions was determined by the Folin–Ciocalteu method. Initially, a diluted Folin–Ciocalteu (FC) solution was freshly prepared in distilled water at a ratio of 1:3, alongside a 3.8% CaCO_3_ solution prepared the previous day. Subsequently, 100 µL of the extraction (100 mg of the residue) was mixed with 2 mL of the FC solution and 7.9 mL of the CaCO_3_ solution. Following a 2 h incubation period in darkness, absorbance was measured at 760 nm using a UV-2600i UV-VIS spectrophotometer (Shimadzu, Kyoto, Japan). A blank control was established using water. Gallic acid served as standard, and results were expressed in mg of GAE/g (gallic acid equivalent per gram) of residue.

#### 2.6.2. Antioxidant Activity Determination (DPPH Method)

The antioxidant potential of the banana leaf powder residues, the extractions, and the impregnated knits (10 days after impregnation) was evaluated using the DPPH method, adapted from [1]. Briefly, 5 mL of a 0.1 mM DPPH solution in ethanol was added to 50 mg of powdered residue, 125 mg of knits, or 150 µL of each extract. The reaction mixture was placed in the dark for 30 min, after which the absorbance was read in a UV-2600i UV-VIS spectrophotometer (Shimadzu) at 517 nm (*ABS_sample_*). A control using water was also prepared and analyzed (*ABS_control_*). When evaluating the antioxidant activity of the knits, a knit control without treatment was used. The results were expressed in antioxidant activity percentage and obtained using Equation (2):

(2) $$Antioxidant\;activity\;\left(\%\right)=\left(\frac{{ABS}_{control}-{ABS}_{sample}}{{ABS}_{control}}\right)*100$$

#### 2.6.3. Antimicrobial Activity of the Functionalized Hemp Knits

The antimicrobial activity of the functionalized hemp knits was evaluated through ATCC TM-100 standard [14,15]. For the essay, 3 microorganisms were used: *Staphylococcus aureus* (ATCC 35984) for antibacterial analysis, and *Candida Krusei* (ATCC 2159) and *Candida albicans* (ATCC 10231) for antifungal analysis. To quantify the MS2 bacteriophage plate-forming units (PFUs), *E. coli* (ATCC 15597) was used as a host. Trypicase Soy Broth (TBS) and solid Trypticase Soy Agar (TSA) were used as the culture media for *S. aureus*, *C. krusei*, and *C. albicans*, and ATCC 271 liquid and solid medium was used for MS2 bacteriophage. The pre-inocula of *S. aureus*, C. *krusei*, and *C. albicans* were incubated overnight at 37 °C (bacteria) and 30 °C (yeast), in TSB, at 120 rpm. Textile square samples of 6.25 cm^2^ were inoculated with 50 µL of 1 × 10^7^ Colony-Forming Units (CFUs)/mL of *S. aureus* and *C. albicans*, previously washed with sterile Phosphate-Buffered Saline (PBS), or 50 µL of 1 × 10^7^ plate-forming units (PFUs)/mL of MS2 in ATCC 271 medium. The samples were incubated at room temperature (21 °C), 1 h. Then, the samples were washed in 5 mL of PBS solution, and mixed in the vortex for 1 min, and serially diluted 3 times. Each dilution was plated in solid medium. The Petri dishes were incubated for 20 h at 37 °C, 90% humidity for *S. aureus*, and 30 °C, 90%, for *C. albicans*, after which the CFUs or PFUs were counted. A control plate was prepared with 50 µL of 1 × 10^7^ CFUs/mL without textile samples and the results were used as a basis to determine the log reduction.

#### 2.6.4. Evaluation of the Ultraviolet Protection Factor (UPF)

The anti-UV protective effect of the produced knits was evaluated according to the standard AS/NZS 4399:2017 [16]. The ultraviolet radiation UVR (290 nm to 400 nm) transmitted through a specimen was measured using a UV-2000s spectrophotometer from Labsphere, NH, USA, equipped with a xenon flash-lamp. The referenced standard categorizes the UV protection level of the samples according to the rated UPF.

### 2.7. Statistical Analysis

All quantitative experiments were made in triplicate and the results expressed in means of ± standard deviation for n = 3. A statistical analysis of the data was conducted using GraphPadPrism v. 8.0 for Windows (GraphPad Software, San Diego, CA, USA, http://www.graphpad.com). After the evaluation of the normality of the results through the Shapiro–Wilk test, when presenting a normal distribution, the data were analyzed using One-way ANOVA and Tukey’s multiple comparisons test; when not presenting a normal distribution, a nonparametric Kruskal–Wallis test was performed. Differences at *p* ≤ 0.05 were considered to be significant (with * denoting statistical differences for *p* ≤ 0.05; ** denoting statistical differences for *p* ≤ 0.01; *** denoting statistical differences for *p* ≤ 0.001; and **** denoting statistical differences for *p* ≤ 0.0001).

## 3. Results

### 3.1. Banana Leaf Powder Properties and Extraction Methodologies

Prior to the extractions, the banana leaves were received and prepared using different strategies (depicted in Figure 1). The main goal was to assess the feasibility of preserving the banana leaves at −20 °C, as a strategy to maintain their properties and facilitate the by-product storage. In the first approach, the banana leaves were washed and immediately dried after reception (A), and the freezing approach (B) involved freezing the samples at −20 °C, with washing and drying carried out as required. The banana leaves were allowed to dry until a consistent dry weight was achieved, which took an average of 48 h for both conditions. Leaves treated with method (A) resulted in a dry weight of 31.5 ± 0.5%, while those treated with method (B) yielded a dry weight of 23.5 ± 1.5%. Following the drying process, leaves from both strategies underwent grinding to achieve a granulometry of 1 mm. The leaves were ground before the extraction processes, since the grinding of plant by-products reportedly leads to much faster kinetics of extraction and efficiencies. In a work from Ngoc et al., it is reported that the extraction yields obtained from the ground material (using a 1 mm mesh size grinder) were much higher for all extraction modes, which included conventional (infusion) and ultrasonic extractions [17]. Considering the final goal of the herein-presented work, this was also the chosen granulometry.

**Figure 3:**
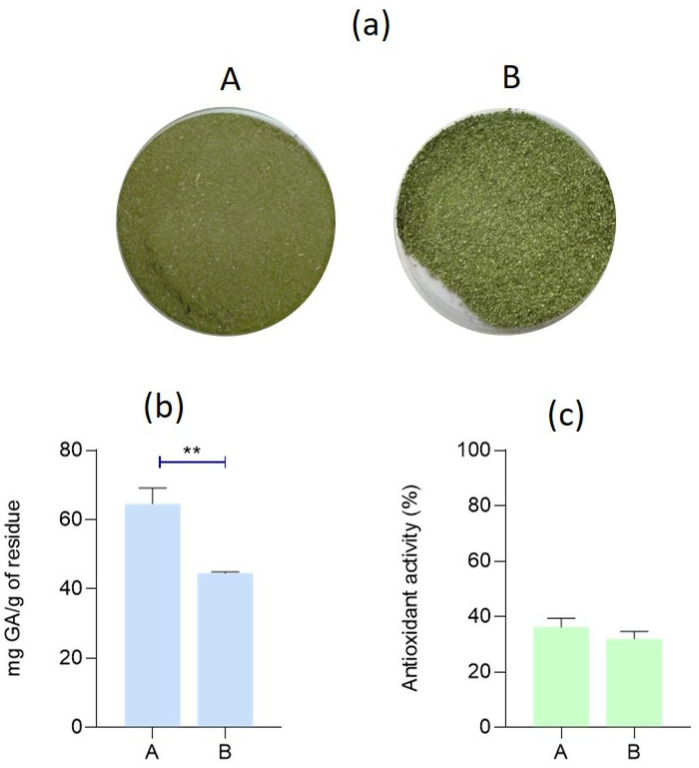
The properties of the leaves stored under different conditions: A—samples were received, washed, and dried; and B—the leaves were received and stored at −20 °C; when needed, they were defrosted, washed, and dried. (**a**) The macroscopic appearance of the banana leaves after grinding; (**b**) the total phenolic content; and (**c**) the antioxidant activity percentage of the banana leaf powder after grinding (where ** denotes statistical differences for *p* ≤ 0.01).

Figure 3 presents the macroscopic appearance of the leaves after grinding (Figure 3a), and the results from the characterization of the banana leaf ground powder in terms of phenolic content and antioxidant activity, obtained following the two preservation strategies upon receiving the leaves, are depicted in Figure 3b and Figure 3c, respectively. Measuring the phenolic content and antioxidant activity of the samples is particularly important in this study, since the distance from the harvest site and transportation time can significantly impact the quality of banana leaves, particularly concerning their phenolic content and antioxidant activity. The findings indicated that employing strategy B led to a small reduction in both phenolic content and antioxidant activity, with the statistical analyses demonstrating significant differences in the total phenolic content. Nevertheless, despite these small differences, we chose to proceed with the extractions from the powder obtained through strategy B, due to logistical constraints preventing us from drying all the leaves simultaneously.

**Figure 4:**
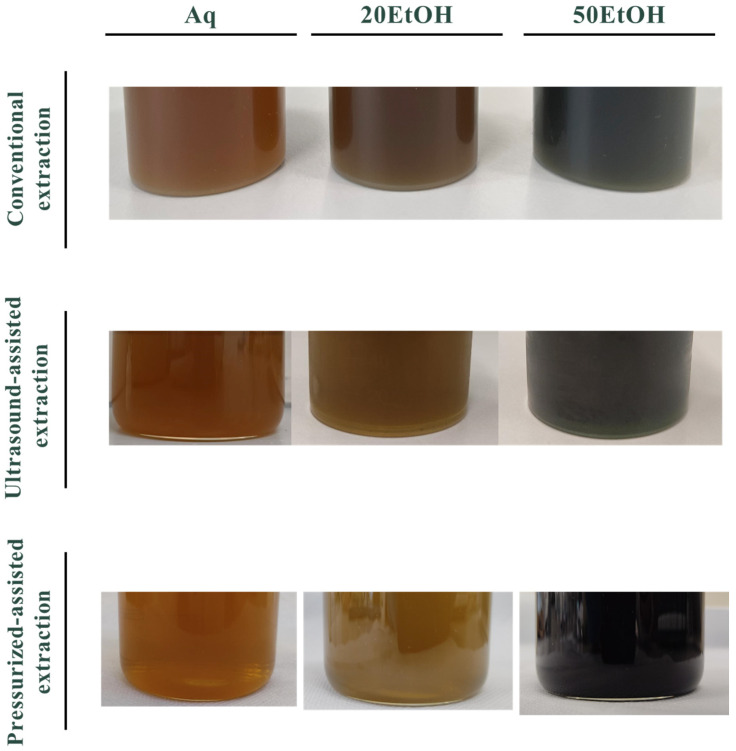
The macroscopic appearance of banana leaf extracts derived from different techniques using both aqueous and hydroethanolic extractions.

Figure 4 depicts the macroscopic appearance of the banana leaf extracts after each extraction process. In general, it was observed that all extracts showed a brownish colour, which is attributed to the drying process of banana leaves. During drying, chlorophyll molecules can undergo chemical reactions, resulting in changes to their structure and colour. These reactions can lead to the loss of magnesium ions from the chlorophyll molecule, causing the pigment to shift from green to a more brownish colour, as observed in the extracts obtained [18,19]. Furthermore, different colours and shades were obtained depending on the solvents and extraction technologies utilized. Extractions performed using water or lower ethanol concentrations (20% (*v*/*v*) EtOH) yielded extracts with a brownish hue. In contrast, extractions conducted with 50% ethanol produced extracts with a strong greenish coloration [20].

To facilitate the interpretation of the results and further discussions, Table 1 delineates the identifications used for the extraction methodologies in the manuscript.

**Figure 5:**
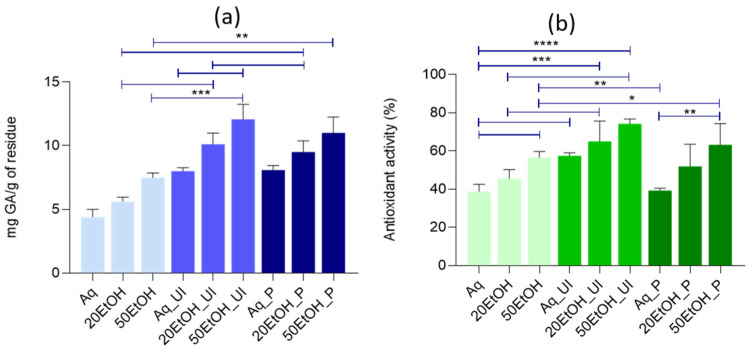
(**a**) The total phenolic content of the banana leaf extracts and (**b**) antioxidant activity percentage. Differences at *p* ≤ 0.05 were considered to be significant (where * denotes statistical differences for *p* ≤ 0.05; ** denotes statistical differences for *p* ≤ 0.01; *** denotes statistical differences for *p* ≤ 0.001; and **** denotes statistical differences for *p* ≤ 0.0001).

The extracts obtained using the different strategies were characterized based on their total phenolic content and antioxidant potential, and the results are presented in Figure 5a and Figure 5b, respectively.

Regarding the total phenolic content, an increase in phenolic concentration was observed with higher ethanolic concentrations, with the maximum concentration achieved using the 50% hydroethanolic media, for all extraction technologies used. This phenomenon can be attributed to the chemical properties of phenolic compounds in banana leaves, which exhibit higher solubility and extraction efficiency in less polar solvents, such as 50% ethanol, when compared with water. When comparing extraction technologies, it is evident that both pressurized (P) and ultrasound-assisted (Ul) extraction significantly increased the total phenolic content compared to conventional extractions (*p* < 0.01 and *p* < 0.001, respectively). The optimal setting for maximizing phenolic content was found for the utilization of ultrasound with 50% hydroethanolic solvent, yielding approximately 12 mg GA/g [21].

Regarding the antioxidant activity (Figure 5b), like the phenolic content, an increase in ethanol concentration resulted in higher antioxidant activity values. Additionally, it is possible to conclude that the aqueous extraction was the least effective solvent for all the methodologies applied, even when using assisted technologies. Concerning the extraction methodologies, there are no significant statistical differences between the pressurized-assisted extraction method and conventional extraction. However, the use of ultrasound shows a marked difference, having a positive impact on antioxidant activity and standing out as the best methodology. Overall, the methodology that resulted in a higher percentage of antioxidant activity was the 50% hydroethanolic extraction using ultrasound, achieving a percentage of nearly 80%, thus showing the possible potential use of such a method to elevate the prospect demonstrated by this residue.

Moreover, and comparing both analyses, TPC and antioxidant activity, is possible to extrapolate a correlation between the antioxidant activity and the total phenolic content, where an increase in one’s percentage led to a similar behaviour on the other assay. Such an analysis is present for every concentration and method, except in the pressurized system, thus depicting the necessity of further analyses to fully understand its potential.

### 3.2. Knits’ Functionalization

Hemp knits were produced (at lab scale using a Tricolab system) using bleached hemp yarn (Ne 15.7). As previously described, the knits were first pre-treated using a natural polymer. The main goal of this is to enhance the durability of the further functionalization steps employing banana leaf extracts, while ensuring that the sustainable features of the process are kept. Usually, metallic mordants are employed to boost the attachment of natural dyes and materials to textile substrates [9,22]. However, this approach might disrupt the sustainability. To overcome this major issue, several works already describe the successful use of natural mordants, including natural-origin polymers [22,23,24].

**Figure 6:**
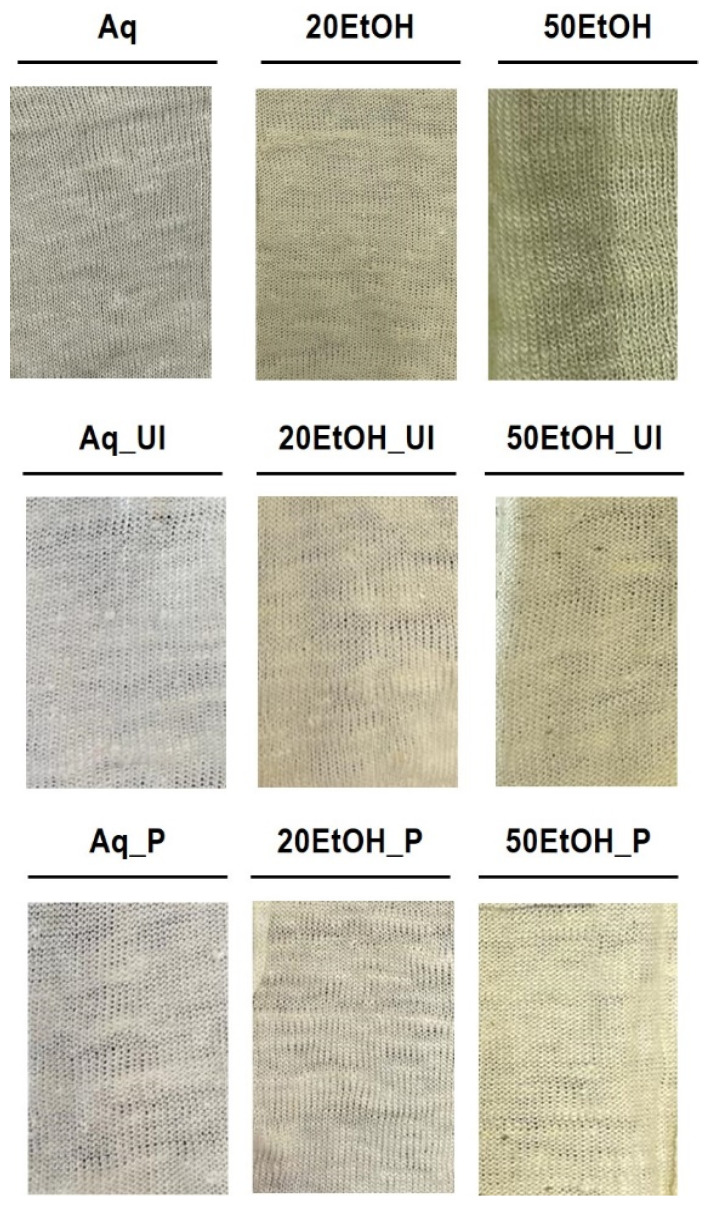
The macroscopic appearance of the knits after functionalization.

Following the pre-treatment, the knits were functionalized using the previously obtained banana leaf extracts described and thoroughly characterized in the previous section. The macroscopic appearance of the knits, after functionalization, is presented in Figure 6. From our analysis, it can be deduced that the percentage of EtOH used in the extraction process significantly influences the colour of the functionalized knits. Specifically, employing a higher percentage of EtOH (50% (*v*/*v*) EtOH) results in knits with a greener hue. This observation aligns with the results obtained for the extracts (Figure 3a), wherein a higher ethanol percentage resulted in a vibrant greener colour. Extractions with a lower ethanol concentration (20% (*v*/*v*) EtOH) yielded extracts with a brownish hue. Consequently, the knits functionalized with these extracts also displayed a light brownish coloration. This is due to the better preservation of chlorophyll in higher ethanol concentrations, which is responsible for the vibrant green colour [20].

**Figure 7:**
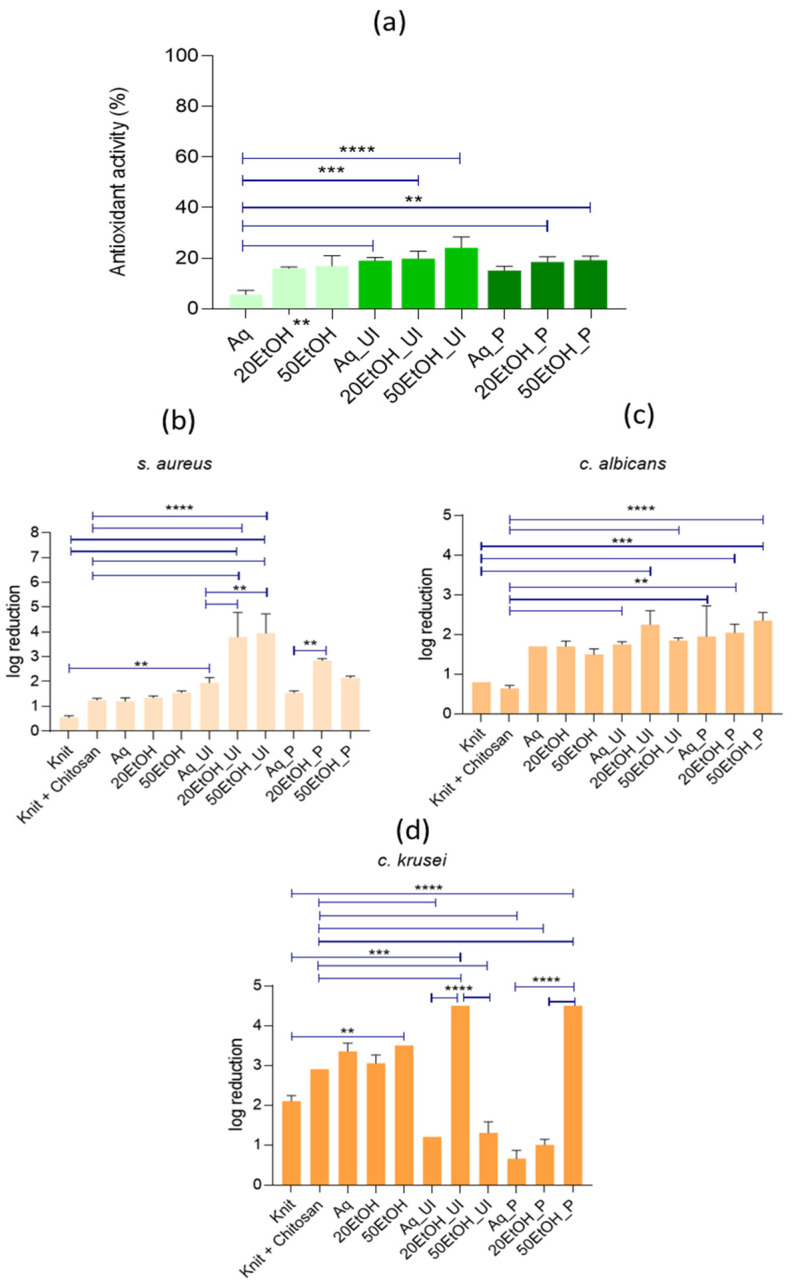
(**a**) The antioxidant activity percentage, where differences at *p* ≤ 0.05 were considered to be significant. The antimicrobial properties of the functionalized and non-functionalized knits against (**b**) *S. aureus*, (**c**) *C. albicans*, and (**d**) *C. krusei*, (where ** denotes statistical differences for *p* ≤ 0.01; *** denotes statistical differences for *p* ≤ 0.001; and **** denotes statistical differences for *p* ≤ 0.0001).

The antioxidant activity analysis of the functionalized knits was also evaluated (Figure 7). The results revealed that, across all extraction methodologies—conventional, ultrasound, and pressurized-assisted—the knits functionalized with the aqueous extract exhibited the lowest percentage of antioxidant activity. This aligns with the antioxidant activity values obtained for the extracts (Figure 5b). Furthermore, and following the previously obtained results for the analysis of the extractions, the hemp knits treated with extracts obtained through ultrasound-assisted methodology exhibited the most promising outcomes when assessing their antioxidant activity, whether with aqueous or hydroethanolic extractions, with no significant differences among the conditions. Statistically significant differences were observed among the knits functionalized using the extracts from conventional aqueous extractions, Aq, and the knits functionalized using extracts from Aq_Ul. Regarding the EtOH concentration, the results indicate that higher concentrations in the extraction process led to higher antioxidant activity values in the functionalized knits. This trend supports the findings from the extract analysis, where extracts obtained with higher EtOH concentrations demonstrated greater antioxidant activity. However, it is important to note that in the case of the functionalized knits, the differences in antioxidant activity between the various ethanol concentrations were not statistically significant. This suggests that while there is a general trend of increased antioxidant activity with higher ethanol concentrations, the variations are not pronounced enough to be considered substantial under the tested conditions for the functionalized knits.

The antimicrobial activity of the functionalized and non-functionalized knits was assessed, and the results are illustrated in Figure 7b–d. These figures demonstrate that the functionalized materials exhibited antimicrobial properties, particularly against Gram-positive bacteria (*S. aureus*) and yeast (*C. krusei*). Notably, the samples treated with the hydroalcoholic extract, namely 20EtOH and 50EtOH, obtained through pressurized method and ultrasound-assisted, respectively, showed the highest antimicrobial activity. This sample was classified as a moderate disinfectant against *S. aureus* and *C. krusei*, and a strong decontaminant against *C. albicans*. The statistical analysis showed that the functionalization of knits using hydroethanolic extracts, particularly using 50% (*v*/*v*) of ethanol concentration with ultrasound-assisted (50EtOH_Ul) or pressurized methods (50EtOH_P), consistently demonstrated significantly higher log reductions compared to other treatments. For *S. aureus*, the 50EtOH_Ul treatment achieved the greatest microbial reduction. Similarly, for *C. albicans*, 50EtOH_Ul showed the highest efficacy, followed closely by 50EtOH_P, with high statistical significance relative to knits functionalized with extracts from conventional extraction methods. In the case of *C. krusei*, 50EtOH_P was particularly effective, significantly outperforming other treatments. Across all microbial species, the controls (knit or knit + chitosan) showed the lowest efficacy, with minimal log reductions and no significant differences when compared to each other. These findings highlight the improved antimicrobial properties of the functionalized knits, particularly those treated with hydroethanolic extracts.

In this study, the UV protection properties of the knits were tested on the non-functionalized (knit) and pre-treated (knit+chitosan) samples, both used as controls, and on functionalized knits (Aq, 20EtOH, 50EtOH, Aq_Ul, 20EtOH_Ul, 50EtOH_Ul, Aq_P, 20EtOH_P, 50EtOH_P), and the results are presented in Table 2. An increase in the Ultraviolet Protection Factor (UPF) was observed across all the functionalized knits compared to both the knit and knit + Pol1 conditions. The knits from the 50EtOH_Ul condition, which previously yielded superior antioxidant activity results (Figure 7a), are regarded as the most effective, as they approach the minimum protection level (UPF = 15) with a UPF value of 13.7 ± 3.1. Knits treated with extracts from ultrasound-assisted extraction exhibited higher UPF values. This observation aligns with earlier data (Figure 5b and Figure 7a), as both the extracts and the knits treated under these conditions showed higher percentages of antioxidant activity. However, according to the standard AS/NZS 4399:2017, all knits presented an ultraviolet protection rating of 0, and further studies will include the optimization of this feature.

## 4. Discussion

### 4.1. Banana Leaf Preservation Strategies

Different strategies were investigated in this study to determine the most effective approach for preserving the banana leaves. Factors such as distance from the harvest site in Thailand and transportation time can significantly influence their quality, especially regarding their phenolic content and antioxidant activity. Additionally, the shipment time—up to four weeks in this case—can still have an impact, despite being conducted under controlled atmospheric conditions with proper ventilation and refrigeration. After harvesting, natural biochemical changes begin to occur, potentially leading to the degradation of sensitive compounds like phenolics and antioxidants. Longer transit times or suboptimal storage conditions can accelerate this degradation, reducing the leaves’ functional qualities, such as their antimicrobial, antioxidant, and preservative properties. Herein, the banana leaves were transported under vacuum sealing and refrigeration, as a strategy to maintain the leaves’ biochemical integrity and maximize their efficacy as a source of bioactive compounds [25]. Additionally, evaluating the phenolic content and antioxidant activity of a residue before extraction is essential for assessing its potential as a source of bioactive compounds and guiding the extraction process for further functionalization. This initial analysis provides a baseline to determine the efficiency of the extraction and helps optimize resources by identifying residues with significant value. It also informs the choice of suitable extraction methods or solvents. Ultimately, this step sets the foundation for efficient extraction and functionalization, ensuring that the process is aligned with intended applications, to provide textiles with antioxidant acidity. Moreover, two different preservation strategies were studied, namely strategies A and B (Figure 1). The primary objective of this preliminary study was to evaluate whether storing the samples at −20 °C and defrosting them as needed (A) or drying and processing them immediately upon receipt (B) would impact the phenolic content of the byproduct and, consequently, its antioxidant activity. The byproducts obtained from these different methodologies were characterized, and the results showed that using strategy B resulted in a slight reduction in both phenolic content and antioxidant activity. Despite these minor differences, we opted to proceed with the extractions from the powder produced by strategy B due to logistical constraints that made it impossible to dry all the leaves at once. Furthermore, considering potential industrial applications, this strategy allows for the preservation of the leaves through freezing, which facilitates a controlled and gradual adaptation process for large-scale implementation.

### 4.2. Comparison of Extraction Methodologies for Maximizing Phenolic Compounds and Antioxidant Activity in Banana Leaf Extracts

Different extraction approaches were studied to fully enhance the potential of banana leaves as a source of phenolic compounds and antioxidant activity. In this study, as mentioned earlier, we investigated the utilization of aqueous and hydroethanolic extraction solvents, varying in ethanol concentrations (20% (*v*/*v*) and 50% (*v*/*v*)), employing both conventional extraction techniques and assisted methods such as ultrasound and pressurized systems. The selected solvents, particularly the hydroethanolic solutions, were chosen based on their ability to cover a wide range of polarities, which is crucial for the extraction of phenolic compounds. Ethanol effectively dissolves a wide range of polar phenols, while hydroethanolic mixtures enhance the solubility of both hydrophilic and moderately hydrophobic phenolic compounds [26]. This combination allows for optimized extraction, as varying ethanol concentrations can selectively target specific phenolic compounds. Herein, low ethanol concentrations and water were studied to improve the cost-effectiveness of the process, to enable further scaling-up. The extraction methodologies utilized included conventional extraction, ultrasound-assisted extraction, and pressurized-liquid extraction. Ultrasound-assisted extraction was chosen for its ability to enhance mass transfer and extraction efficiency through cavitation effects, allowing for a faster and more effective extraction of bioactive compounds, particularly those that are heat-sensitive [27,28,29]. Although all methods were conducted at 50 °C for 30 min to maintain consistency, ultrasound-assisted extraction typically allows for lower temperatures, making it advantageous for preserving sensitive compounds. Pressurized-assisted extraction was included for its capacity to utilize high pressure, thereby increasing the solvent’s extraction efficiency [30]. The standardized temperature and reaction time across all extraction methods ensured the comparability of results, minimizing the variability that could influence the outcomes. By employing a diverse array of techniques, this study aimed to maximize the extraction yield and diversity of compounds from the source material. It is known that assisted technologies, particularly ultrasound and pressurized systems, consistently outperformed conventional ones in terms of extraction yield and extraction rate. Despite the proven effectiveness of assisted technologies [31], conventional methods are still commonly tested and utilized since they are often simpler, requiring less specialized equipment and expertise, which makes them more accessible and cost-effective.

Afterward, differences in the colour of the extracts were noticed, as mentioned previously. These alterations may be attributed to the affinity and stability of chlorophyll (responsible for the green colour) for more organic solvents such as the 50% EtOH [20]. Upon comparison of the different technologies herein explored, it is evident that extracts obtained via conventional and ultrasound-assisted extraction exhibit similar colorations. In contrast, those obtained via pressurized-liquid extraction demonstrate a lighter coloration, which could indicate the lower efficiency of this approach.

Regarding the characterization of the banana leaf extracts, and in terms of phenolic content, slight variations from that reported the literature are noticeable. Other studies, such as in the case of Schmidt et al. on banana inflorescences, have reported a higher phenolic content obtained through conventional extraction using 50% (*v*/*v*) EtOH (aq.) with a 30 min extraction at 60 °C, compared to ultrasound-assisted extraction. In this case, the authors reported a value of nearly 16.9 mg GA/g, while in this study, for similar conditions, a value of 8 mg GA/g was recorded [21]. The discrepancy in the results and the superior outcomes achieved with ultrasound in this study could be attributed to the differing types of banana residue studied and the variations in the ultrasound technologies employed. Whereas in this study the ultrasound is used as a pre-treatment for the residue, in the case of the work developed by Schmidt et al., the ultrasound is used simultaneously as the extraction is happening.

Additionally, and through both analyses, it was possible to understand the efficiency of ethanol in increasing the amount of compounds of interest. This aligns with previous studies, which also showed that aqueous extraction yielded less efficient results, because ethanol is an effective solvent for extraction processes, being preferred over water [32]. In addition, the steady increase in the ethanol percentage is related to an equal rise in antioxidant activity. As described, this might be related to the increase in solvent polarity and the affinity of the compounds present in banana leaves for those types of solvents [18,19]. Concerning the methodologies used, ultrasound-assisted extraction presented the highest percentage of antioxidant activity, as well as the phenolic content. On the contrary, in the literature, the use of ultrasound is depicted as having a negative or similar impact on the extraction of some compounds from natural products, since the energy liberated from the equipment can transform the molecules of interest [21,33,34]. The use of ultrasound is also reported to facilitate the mass transfer and the breakage of the cell membrane in the solution, thus improving the extraction of compounds of interest [27,29]. Further investigation would aid in fully understanding the effects of such techniques, and how differences in usage could help unlock the full potential of residues as a means to improve the sustainability of specialized textiles.

Lastly, a correlation was noticeable between the phenolic content and the antioxidant activity, where an increase in one lead to an equal increment on the other. This association between both categories is reported in the literature, where the content of phenolics can be responsible for the antioxidant performance of the banana leaves [21].

### 4.3. Antimicrobial and UV-Protective Properties of Functionalized Hemp Knits

According to what is reported in the literature, hemp possesses natural antibacterial properties that boost the interest in hemp knits, but also influence the assays described in this article [35]. In this study, chitosan is utilized as a pre-treatment because of its cationic properties, which make it an effective textile additive and finishing agent for enhancing colour and functional durability, as is widely reported [23,36,37]. Also, chitosan has been documented in numerous studies as a polymer used to produce eco-friendly antimicrobial textiles [23,36,37]. In line with previous studies, the present research demonstrates that the pre-treated knits (knit + chitosan) exhibit improved antimicrobial properties compared to the original knits (knit), likely due to the presence of chitosan. The functionalized samples exhibited log reductions ranging from 1 to 4.5, categorizing them from weak decontaminants to moderate disinfectants [38]. Moreover, a log reduction greater than 3 represents the division between decontaminant and disinfectant, while a log superior to 6 demonstrates the sterilization capacity of the agent in question. Thus, a log reduction closer to these thresholds indicates stronger decontaminants or weaker disinfectants depending on if they are above or under the limit indicated prior [35,39,40].

The antimicrobial efficacy of the banana leaf extracts, obtained in this study, can be attributed to the presence of phenolic compounds, especially polyphenol oxidase, that was already reported in the literature [41]. As indicated in Figure 5a, extracts with higher phenolic content were obtained through ultrasound-assisted extraction. However, samples functionalized with extracts obtained via a pressurized system exhibited equal or even greater antimicrobial activity, particularly against *S. aureus*. This suggests that the method of extraction influences the antimicrobial properties of the extracts, as different phenolic compounds can be obtained through different methods. Furthermore, samples functionalized with hydroalcoholic extracts displayed higher antimicrobial activity compared to those functionalized with aqueous extracts. The higher phenolic content in hydroalcoholic extracts is likely responsible for this increased antimicrobial efficacy [42,43,44].

Regarding UV protection, several published works have investigated the effectiveness of utilizing natural fibres combined with natural bioactive components as an effective method to minimize health risks from the current levels of UV radiation on Earth’s surface [11,45]. These compounds were presented as promising resources for protection from the sun due to their ability to absorb UV radiation and their antioxidant properties [46]. Briefly, phenolics, abundantly present in the extracts of natural plants, including the ones from banana leaves investigated in the present study (Figure 5a), are believed to play a role in redox-sensitive signalling pathways, potentially preventing DNA damage and countering the generation of oxygen-free radicals and lipid peroxidation induced by UV exposure. Given these implications, the antioxidant activity of phenolics is crucial for effective UV protection [46].

## 5. Conclusions

Herein, knitted hemp fabrics with antimicrobial properties were successfully developed, using a sustainable and easily scaled-up method by combining green processing methods and naturally derived antimicrobial compounds. The assisted extraction methods, namely ultrasound-assisted extraction, yielded greater results from the banana leaf residues, with higher ethanol concentrations (50EtOH) producing greener extracts with higher phenolic content and antioxidant activity, ultimately leading to knitted fabrics with higher antioxidant and antimicrobial properties. Hemp knits pre-treated with a natural polymer and functionalized with the ultrasound-assisted extracts showed colour variations dependent on ethanol concentration, with 50% ethanol providing greener knits. Antioxidant activity was highest in knits treated with ultrasound-assisted extracts using 50EtOH as the extraction solvent, while the antimicrobial properties were most pronounced in knits treated with hydroalcoholic extracts obtained from pressurized methods. These extracts enhanced UV protection, with knits treated with 50% ethanol extracts via ultrasound-assisted extraction showing the highest UV Protection Factor (UPF). Overall, higher ethanol concentrations and ultrasound-assisted extraction significantly improved the functional properties of banana leaf extracts, enhancing the functional properties of hemp knits, and underscoring their sustainable potential for textile applications. In general, the best condition for developing functionalized hemp knits appears to be using ultrasound-assisted extraction with 50% ethanol (50EtOH). This condition produced extracts with the highest phenolic content and antioxidant activity, resulting in greener knits with enhanced antimicrobial properties and the highest UV Protection Factor (UPF), leading to superior performance in the resulting knitted hemp fabrics. However, it requires further optimization to achieve greater durability and ensure scalable results for industrial applications.

The development of environmentally friendly specialized textiles with antimicrobial properties is becoming vital, mostly due to the environmental risk associated with the careless disposal of textiles with organic and mineral antimicrobial finishings. The proposed strategy provided insights toward the development of innovative functional textiles, with enhanced bioactive properties, suitable for a wide range of applications in both textile and healthcare fields.

## Figures and Tables

**Table 1:** The identifications of the extraction methodologies used in the manuscript.

Extraction	Solvent	Sample ID
Conventional extraction	Aqueous	Aq
	Hydroethanolic-20% EtOH	20EtOH
	Hydroethanolic-50% EtOH	50EtOH
Ultrasound-assisted extraction	Aqueous	Aq_Ul
	Hydroethanolic-20% EtOH	20EtOH_Ul
	Hydroethanolic-50% EtOH	50EtOH_Ul
Pressurized-liquid extraction	Aqueous	Aq_P
	Hydroethanolic-20% EtOH	20EtOH_P
	Hydroethanolic-50% EtOH	50EtOH_P

**Table 2:** Ultraviolet Protection Factor (UPF) of the hemp knits.

Sample ID	Ultraviolet Protection Factor (UPF)
Knit	4.6 ± 0.52
Knit + Chitosan	5.2 ± 0.64
Aq	11.6 ± 3.3
20EtOH	9.8 ± 0.71
50EtOH	8.2 ± 2.9
Aq_Ui	12.3 ± 2.7
20EtOH_Ul	12.0 ± 3.5
50EtOH_Ul	13.7 ± 3.1
Aq_P	10.9 ± 0.71
20EtOH_P	6.7 ± 0.61
50EtOH_P	10.9 ± 1.0

## Data Availability

The original contributions presented in the study are included in the article, further inquiries can be directed to the corresponding author.
